# Insulin Signaling and Insulin Resistance Facilitate Trained Immunity in Macrophages Through Metabolic and Epigenetic Changes

**DOI:** 10.3389/fimmu.2019.01330

**Published:** 2019-06-12

**Authors:** Eleftheria Ieronymaki, Maria G. Daskalaki, Konstantina Lyroni, Christos Tsatsanis

**Affiliations:** ^1^Laboratory of Clinical Chemistry, University of Crete Medical School, Heraklion, Crete, Greece; ^2^FORTH, Institute of Molecular Biology and Biotechnology, Heraklion, Crete, Greece

**Keywords:** macrophages, metabolism, epigenetic, Akt, mTOR, trained immunity

## Abstract

Adaptation of the innate immune system has been recently acknowledged, explaining sustained changes of innate immune responses. Such adaptation is termed trained immunity. Trained immunity is initiated by extracellular signals that trigger a cascade of events affecting cell metabolism and mediating chromatin changes on genes that control innate immune responses. Factors demonstrated to facilitate trained immunity are pathogenic signals (fungi, bacteria, viruses) as well non-pathogenic signals such as insulin, cytokines, adipokines or hormones. These signals initiate intracellular signaling cascades that include AKT kinases and mTOR as well as histone methylases and demethylases, resulting in metabolic changes and histone modifications. In the context of insulin resistance, AKT signaling is affected resulting in sustained activation of mTORC1 and enhanced glycolysis. In macrophages elevated glycolysis readily impacts responses to pathogens (bacteria, fungi) or danger signals (TLR-driven signals of tissue damage), partly explaining insulin resistance-related pathologies. Thus, macrophages lacking insulin signaling exhibit reduced responses to pathogens and altered metabolism, suggesting that insulin resistance is a state of trained immunity. Evidence from Insulin Receptor as well as IGF1Receptor deficient macrophages support the contribution of insulin signaling in macrophage responses. In addition, clinical evidence highlights altered macrophage responses to pathogens or metabolic products in patients with systemic insulin resistance, being in concert with cell culture and animal model studies. Herein, we review the current knowledge that supports the impact of insulin signaling and other insulin resistance related signals as modulators of trained immunity.

## Introduction

Obesity induces metabolic inflammation, a chronic low-grade inflammation characterized by systemic increased levels of pro-inflammatory factors and insulin resistance. Energy storage organs like adipose tissue, liver and muscle exceed their capacity to preserve homeostasis, leading to metabolic stress. this condition is capable of altering responses mediated by innate immune cells. Macrophages are central mediators of inflammatory responses and are causally linked to obesity-related pathologic conditions, including Type 2 diabetes (T2D) and cardiovascular disease (1).

During recent years, the dogma that only adaptive immunity is responsible for immune memory has been challenged and the concept of innate immune training has emerged. In plants, invertebrates and mammals, cells of innate immunity have been shown to possess a form of memory, also termed “Trained Immunity.” Trained immunity is defined as persistent alteration of innate immune responses, which depends on prior exposure to a signal. For example, exposure to β-glucan, infection or vaccination, results in elevated pro-inflammatory cytokines after a secondary infection. Exposure to insulin or Saturated Fatty Acids (SFAs) also results in altered TLR responses (2, 3) Trained immunity also describes altered responses to pathogens occuring after severe inflammation, such as endotoxin tolerance or Compensatory Anti-inflammatory Response Syndrome (CARS) (4). Innate immune cells shown to be part of “trained immunity” are monocytes, NK cells and dendritic cells (5, 6). This review will focus on macrophage trained immunity, which includes bone marrow-derived monocytes/macrophages and tissue resident macrophages that are self-renewing embryo-derived (7–10). Innate immune training is not only associated with infectious stimuli but also to non-infections conditions, such as obesity and insulin resistance (3, 11, 12).

**Figure 1:**
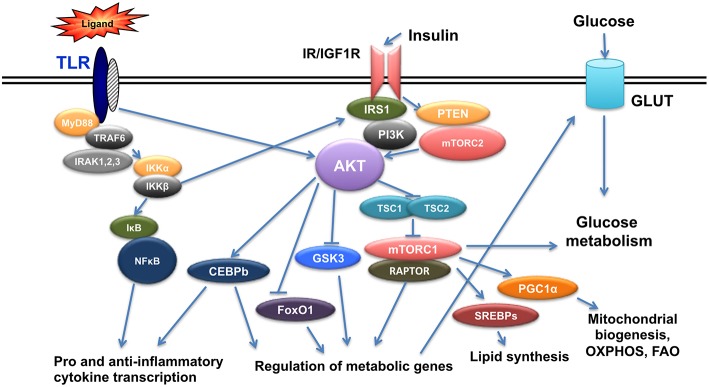
Crosstalk of TLR and Insulin signals. TLR triggering initiates a cascade of events that results in activation of NFkB and AKT leading to pro- and anti- inflammatory gene expression as well as genes regulating metabolism. Insulin signals also activate AKT and result in regulation of metabolic gene expression and at the same time crosstalk with TLR signaling.

Insulin is a hormone produced from pancreatic beta cells to regulate glucose homeostasis, fat metabolism and cell growth. Insulin levels change in the context of diabetes and it is now established that insulin signals in immune cells modulate their function (13–15). Insulin mediates its signal through insulin receptor (IR), but also through its highly homologous Insulin-like growth factor 1 receptor (IGF1R). Binding of insulin on IR or IGF1R results in the phosphorylation of insulin receptor substrate 1/2 (IRS1/2) at its tyrosine residues and in the subsequent activation of two main pathways, the Phosphoinositide3-kinase(PI3K)/AKT pathway and the mitogen activated protein kinase (MAPK) pathway (16, 17). AKT is a family of serine/threonine kinases encoded by three highly homologous genes (Akt1, Akt2, and Akt3) (18). It is phosphorylated by phosphoinositide-dependent protein kinase-1(PDK1) and mammalian target of rapamycin complex 2(mTORC2) to regulate glucose transporters (Glut), glycogen synthase kinase 3(GSK3), forkhead box O1 transcription factor (FoxO1), and mammalian Target of Rapamycin Complex 1 (mTORC1), and involved in glucose uptake, glycogen synthesis, and protein synthesis. Activation of mTORC1 from AKT is mediated through Tuberous Sclerosis-2(TSC2) phosphorylation and inactivation. TSC2 is a tumor suppressor that forms a complex with TSC1 and inactivation of the TSC1/TSC2 complex activates mTORC1 with its specific protein Raptor (19–21) (Figure 1).

In this review we present current knowledge on insulin signaling in innate immune cells focusing on macrophages and how changes in insulin signaling affect their responsiveness and provide a form of innate immune training.

### Insulin Signaling Modulates TLR Responses in Macrophages Facilitating Trained Immunity

During obesity, insulin signaling pathway is deregulated and a state of low grade systemic inflammation is established. Macrophages react to both TLR and insulin signals shaping their responses. Hyperlipidemia and increased levels of SFAs can trigger inflammation through direct binding to TLR2 and TLR4 receptors (22). SFAs promote inflammation through metabolic reprogramming of macrophages resulting in activation of mTOR and lipid metabolism *in vivo* altering TLR4-dependent signals (2), thus promoting training of macrophages. Priming of macrophages can also occur by circulating bacterial endotoxin originating from altered gut microbiome due to increased intestinal permeability occurring in obesity (23). In addition, increased levels of free fatty acids results in incomplete metabolism and accumulation of fatty acid intermediates, like diacylglycerol (DAG) and ceramides that are able to activate several protein kinases in macrophages such as Protein Kinase C (PKC), Janus Kinase (JNK), and IκB Kinaseβ (IKKβ) resulting in subsequent inactivation of IRS-1 and thus inhibition of insulin signaling both in culture and *in vivo* (24). Increased insulin, amino acids and pro-inflammatory cytokines due to chronic overnutrition in the context of obesity activate mTORC1 that mediates feedback inhibition to PI3K/AKT pathway (21). AKT signals also regulate CCAAT-Enhancer-Binding Protein b(CEBPb), a transcription factor that controls anti-inflammatory gene expression and expression of the TLR signaling regulator IRAK3 (25, 26) (Figure 1). Thus, signals initiated by metabolic factors modulate molecules that control TLR responses.

Type 2 diabetes and obesity result in hyperglycemia, which readily impacts macrophage responses, providing training in macrophages. Advanced glycation end products (AGEs), glycated proteins or lipids formed non-enzymatically due to increased glucose concentration are recognized by receptor of AGEs (RAGE). Stimulation of RAGE results in increased ROS production and subsequent NF-kB activation (27). In addition to oxidative stress, endoplasmic reticulum (ER) stress is also induced in obesity. Stimulation of Unfolded Protein Response(UPR) signaling pathways as a consequence of ER stress results in activation of protein kinases IKKβ and JNK (28, 29), also impacting TLR signaling. Adipokines promote innate immune training, such as adiponectin that suppresses TLR4 responses via induction of IRAKM in culture and *in vivo* (25, 30). Adiponectin also enhances insulin sensitivity via IRS2 (31, 32). Finally, cytokines released by activated immune cells further contribute to inflammation and impairment of insulin signaling in macrophages, altering innate immune responses (33).

### Changes in Cell Metabolism Shape Macrophage Training in the Context of Insulin Signaling

Macrophage metabolism is tightly linked to their capacity to respond to TLR signals and characterizes their polarization status. M1 activation of macrophages, the state of activated pro-inflammatory macrophages, is characterized by increased rates of glycolysis, induction of Pentose Phosphate Pathway (PPP), arginine conversion to nitric oxide (NO) by iNOS and fatty acid synthesis and oxidation, essential for pro-inflammatory signaling and NLRP3 inflammasome activation. Anti-inflammatory macrophages, broadly termed as M2-type, display increased glutamine uptake and catabolism, arginine metabolism by arginase-1, oxidative phosphorylation, enhanced fatty acid oxidation and glycolysis. The importance of glycolysis for development of M1-phenotype is supported by the fact that deletion of Solute Carrier2A1 (Slc2A1), which encodes Glucose Transporter1 (GLUT1), results in reduced LPS responses and M2-like phenotype (34), while its overexpression enhanced response to LPS and Reactive Oxygen Species (ROS) production (35). Upon M1 activation, expression of Fatty Acid Transporter1 (FATP1) is reduced promoting glycolysis, as demonstrated in *Fatp1*^−/−^ or FATP1-overexpressing macrophages (36). GLUT1 expression controls efferocytosis and removal of apoptotic debris. At the same time Solute Carrier16A1 (Slc16A1) facilitates lactate release, the by-product of aerobic glycolysis known to promote M2-type polarization (37). Glutamine metabolism is also important for the propagation of epigenetic modifications evident in trained macrophages (38). Therefore, changes in macrophage metabolism are tightly linked to their polarization status and training.

During obesity, Adipose Tissue Macrophages (ATMs) acquire a unique metabolic profile not characteristic of M1-type or M2-type activation displaying both increased glycolysis and oxidative phosphorylation. Glycolysis was found to regulate the secretion of pro-inflammatory factors (39). In the context of obesity, peripheral macrophages also become resistant to insulin exhibiting increased glycolysis, important for the regulation of their M2-like polarization status (3). Thus, insulin resistance is associated with a distinct metabolic phenotype in macrophages affecting innate immune responses and therefore contributing to training.

### Insulin Resistance Confers Innate Immune Training and an M2-Like Phenotype

Macrophages express all components of insulin signaling, indicating a functional insulin signaling cascade and develop insulin resistance in the context of systemic insulin resistance (3, 40). Resident macrophages obtain M1 or M2 phenotypes, depending on the microenvironment and the stimuli present, such as this of insulin that induces AKT kinase signals (3, 41). In the context of insulin resistance, peripheral macrophages acquire an anti-inflammatory M2-like phenotype, characterized by increased expression of M2 markers and reduced secretion of pro-inflammatory factors upon stimulation (3, 42). In addition, insulin resistant macrophages produce reduced levels of NO upon TLR stimulation *in vivo* and in culture and exhibit reduced bactericidal capacity *in vivo* (3).

ATMs acquire an M1-type pro-inflammatory phenotype in the inflamed adipose tissue during obesity (43–45). This M1-like phenotype includes expression of both oxidative and glycolytic genes (39, 46). M1-like ATMs express the scavenger receptor CD36, ATP-binding cassette transporter1 (ABCA1) and Perilipin (PLIN) as membrane markers, regulated by Peroxisome Proliferator-activated Receptor-γ(PPARγ), and do not express M1 markers (47).

In contrast to adipose tissue macrophages, M2-like polarized macrophages were also found in other conditions associated with systemic insulin resistance (obesity and type 2 diabetes), such as cancer and cardiovascular disease (48, 49). In cancer, for example, Tumor-Associated-Macrophages (TAMs) possess an M2-like phenotype and support tumor growth (50). In atherosclerosis, foam cells display M2-like properties and are found in atheromatic plaques (11, 51). In animal models, genetic deletion of IR in macrophages results in an anti-inflammatory behavior (52) and protects against diet-induced obesity (53). IGF-1R protein is suppressed in M1-type activated macrophages (54, 55) and genetic ablation of IGF-1R results in suppression of M1 responses and enhanced M2 responses in the context of skin inflammation (42). In addition, myeloid restricted deletion of IGF1R favors an M2-like polarization and protects mice from sepsis and inflammatory bowel disease (3, 56, 57). Moreover, IRS2, a substrate of both IR and IGF1R, suppresses alternative activation of macrophages *in vivo* (58). All the above findings support that inhibition of insulin signals promote an M2-like phenotype in peripheral macrophages.

Deletion of the downstream insulin signaling components, AKT kinases, results in differential polarization status of macrophages. AKT2 is the predominant isoform that participates in insulin signaling cascade (18, 59). Genetic ablation of Akt2 renders macrophages insulin resistant (3) and promotes an M2-like phenotype *in vivo* (56). Indirect activation of mTORC1 from AKT is important in propagating insulin signals. Deletion of Raptor from macrophages, elevates M2 macrophage population in LysM^Cre^Rptor^fl/fl^ mice (60), consistent with the effect of TSC1 deletion, which results in sustained activation of mTORC1 and decrease of M2 population (60, 61). In contrast, lack of TSC2 in macrophages enhanced the expression of M2 polarization markers (62). Insulin resistant macrophages, namely macrophages from obese mice or lacking AKT2, exhibit increased mTORC1 basal activity. Basal mTORC1 is important for their activation status, since treatment with the mTOR inhibitor rapamycin abrogated their M2-like phenotype and at the same time changed their metabolism in culture (3). Hence, deletion of molecules participating in insulin signaling such as AKT2, or the mTORC1 regulators TSC1 or TSC2, revealed the role of insulin signaling in modulating macrophage responses and support the role of insulin signaling in trained immunity.

### Epigenetic Regulation of Trained Immunity and the Role of Insulin Signaling and Metabolism

The molecular basis of trained immunity is mediated by transcriptional and epigenetic reprogramming, based on sustained alteration in gene expression regulated by DNA and histone modifications (63). Information on DNA modifications associated with trained immunity is limited to modifications of the TNF locus in the context of endotoxin tolerance (64), while most of the information available is focused on histone modifications (65). Upon primary stimulation of myeloid cells, a cascade of transcriptional signals ensures tight regulation of inflammatory genes, through recruitment of transcription factors, such as NF-kB, AP-1 and Signal Transducer and Activator of Transcription (STAT) family members, to enhancers and gene promoters. These transcription factors in turn recruit chromatin modifying enzymes to enhance genome accessibility via histone modifications. The persistence of such histone modifications may itself affect secondary responses (8). De novo enhancers acquire histone modifications, such as H3K4me1, only after a primary stimulus (8).

Epigenetic reprograming is mediated by inflammatory signals and lead to histone modifications that alter gene expression patterns (66). Among inflammatory signals that epigenetically reprogram macrophages are NLR Pyrin Domain Containing Protein 3 (NLRP3) signals (6, 67). In addition, trained immunity can be triggered by oxidized low-density lipoprotein (oxLDL) in monocytes. OxLDL is being phagocytosed by macrophages resulting in foam cell formation and atherosclerosis. Exposure of cells to oxLDL leads to trimethylation of lysine 4 at histone 3 (H3K4) in promoter regions of *tnf*α, *il-6, il-18*, the Matrix Metalloproteinase genes *mmp2, mmp9*, and the scavenger receptor cd36, which all contribute to foam cell formation *in vivo* (68). Epigenetic reprogramming induced by oxLDL was found to be regulated through mTOR-dependent oxidative stress (12). In obesity and diabetes, hyperglycemia can trigger “hyperglycemic memory,” characterized by sustained NF-kB gene activity due to epigenetic marks, like increased H3K4 and reduced H3K9 methylation (69). In this context, high glucose levels induce epigenetic changes and training of macrophages (70).

Epigenetic modifications are regulated by both immune signaling and metabolic pathways, since metabolites such as SAM, acetyl-CoA, NAD^+^, and ATP are used as substrates or cofactors for chromatin modifying enzymes (6, 71). As a result, changes in metabolite concentrations regulate gene expression by modulating the epigenome and alter chromatin dynamics (72). For example, α-ketoglutarate produced as an intermediate by the TCA cycle via glutaminolysis promotes alternative activation of macrophages via epigenetic changes in M2 related genes through JMJD3 H3K27 demethylase activity (73).

Fumarate, a key metabolite for the induction of innate immunity, is mediated through induction of H3K4me3 and H3K27Ac epigenetic marks on promoters of genes encoding pro-inflammatory cytokines. Accordingly, fumarate positively regulated the transcription of genes encoding the H3K4 demethylase KDM5 isoforms in culture and *in vivo* (74). As a result, BCG-trained macrophages exhibited enhanced mRNA levels of key glycolysis genes like *hk2* and *pfkp*, the promoter of which acquired more H3K4me3 and less H3K9me3 methylation marks. Therefore, cells displayed a shift toward glycolysis and oxygen consumption and reduced glutamine metabolism (75). In contrast, in β-glucan trained human macrophages, a Warburg effect was observed displaying high glycolytic rate and decreased oxidative phosphorylation (76), supporting the hypothesis that individual training stimuli utilize distinct metabolic programs. For example, mevalonate alone induced trained immunity in macrophages through changes in H3K27ac of inflammatory gene enhancers (77). Thus, elevated glycolysis and the accumulation of the TCA intermediate metabolites fumarate and glutamate, control H3K4me3, and H3K27ac, forming the metabolic basis of essential metabolo-epigenetic pathways mediating trained immunity (78).

### Insulin Resistance and Trained Immunity: A Clinical Perspective

Dysregulated glucose metabolism and overutilization of glucose have been shown to promote inflammation in monocytes and macrophages from patients with atherosclerotic coronary artery disease (CAD) (79). The cause of inflammation originates from nutrient over-supply, glucose overutilization and imbalanced ROS generation (79). It is well-established that monocytes from T2D patients display enhanced activation of the inflammasome (80). As a result the hyperglycemic environment in T2D patients promotes training of monocytes and macrophages. Endogenous metabolic products can also promote immunological training. For example, monocytes derived from humans with high levels of Lipoprotein A(Lpa), a cardiovascular risk factor that carries oxidized phospholipids, have increased capacity of producing pro-inflammatory cytokines upon stimulation and this is phenotype is also observed in macrophages from healthy individuals treated with oxidized LDL (68, 81, 82). Oral supplementation with the metabolic byproduct butyrate, also decreases training of peripheral mononuclear macrophages by oxidized LDL in obese humans with metabolic syndrome (83).

Cancer development has been associated with obesity and insulin resistance. There is evidence that innate immune training initiated by vaccination lowers cancer incidence (84). Treatment with anti-diabetic drugs such as metformin has been linked to lower risk for cancer development and improve outcome in cancer patients (85, 86). The action of insulin sensitizers on cancer is complex since they do not only affect low grade systemic inflammation and cell metabolism that impacts the disease but also directly affect cancer cell homeostasis and survival (85). Nevertheless, utilizing trained immunity as a therapeutic approach in cancer, cardiovascular and infectious diseases has been proposed (87). In this context, insulin sensitization may contribute in training macrophages for therapeutic purposes.

## Conclusions

**Figure 2:**
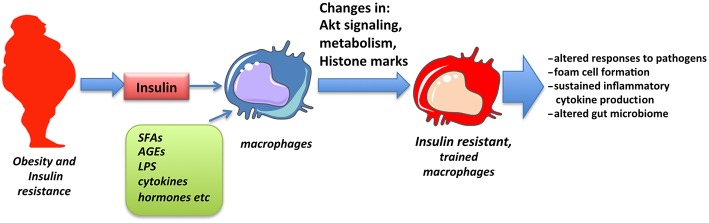
Obesity and insulin resistance promote macrophage training. Macrophages chronically exposed to high levels of insulin as well as SFAs, adipokines, inflammatory cytokines, and low levels of endotoxin, all associated with obesity, obtain changes in Akt signaling, cell metabolism and epigenetic alterations in inflammatory genes that result in altered responses, described as innate immune training.

The concept of trained immunity has emerged in recent years to explain sustained changes observed in innate immune responses following exposure to pathogenic or environmental stimuli. Among those, insulin signaling and insulin resistance has been shown to promote training of cells that may partly explain altered responses and pathologies associated with obesity and insulin resistance (Figure 2). The detailed molecular mechanism through which insulin signaling contributes to trained immunity is yet to be explored.

## Author Contributions

All authors listed have made a substantial, direct and intellectual contribution to the work, and approved it for publication. EI, MGD, KL, and CT drafted the manuscript.

### Conflict of Interest Statement

The authors declare that the research was conducted in the absence of any commercial or financial relationships that could be construed as a potential conflict of interest.
